# Review on Multicatalytic Behavior of Ba_0.85_Ca_0.15_Ti_0.9_Zr_0.1_O_3_ Ceramic

**DOI:** 10.3390/ma16165710

**Published:** 2023-08-21

**Authors:** Akshay Gaur, Chirag Porwal, Imed Boukhris, Vishal Singh Chauhan, Rahul Vaish

**Affiliations:** 1 School of Mechanical and Materials Engineering, Indian Institute of Technology Mandi, Kamand 175005, Himachal Pradesh, India; d20011@students.iitmandi.ac.in (A.G.); d20055@students.iitmandi.ac.in (C.P.); 2 Department of Physics, Faculty of Science, King Khalid University, Abha 62529, Saudi Arabia; imed_boukhris@yahoo.fr

**Keywords:** ferroelectric ceramic, multicatalytic activity, Ba_0.85_Ca_0.15_Ti_0.9_Zr_0.1_O_3_ (BCZTO), composite

## Abstract

Ferroelectric materials are known to possess multicatalytic abilities that are nowadays utilized for removing organic pollutants from water via piezocatalysis, photocatalysis, piezo-photocatalysis, and pyrocatalysis processes. The Ba_0.85_Ca_0.15_Ti_0.9_Zr_0.1_O_3_ (BCZTO) ceramic is one such ferroelectric composition that has been extensively studied for electrical and electronic applications. Furthermore, the BCZTO ceramic has also shown remarkable multicatalytic performance in water-cleaning applications. The present review explores the potentiality of BCZTO for water-cleaning and bacterial-killing applications. It also highlights the fundamentals of ferroelectric ceramics, the importance of electric poling, and the principles underlying piezocatalysis, photocatalysis, and pyrocatalysis processes in addition to the multicatalytic capability of ferroelectric BCZTO ceramic.

## 1. Introduction

Industrial dyes are one of the representative organic contaminants that result in water pollution in terms of both aesthetics and toxicity [1,2]. In addition to endangering humans, contaminated water has a negative impact on marine life as well, which is also a key component of our ecosystem. Catalysis plays a vital role in water-cleaning applications by providing efficient and sustainable methods to remove pollutants, improve water quality, and protect human/environmental health. The development of innovative catalytic materials and processes continues to advance the field of water treatment and contribute to the sustainable management of water resources. Advanced oxidation processes (AOPs) are one of the solutions in water remediation, which comprise a diverse set of oxidation techniques in the aqueous phase that rely on highly reactive species, mainly hydroxyl radicals, to effectively degrade and eliminate targeted pollutants. By harnessing the exceptional reactivity of hydroxyl radicals, these methods initiate intricate oxidation reactions, leading to the breakdown and elimination of various pollutants found in water. While hydroxyl radicals are the primary drivers in AOPs, it is important to acknowledge that other reactive species also contribute to these processes, albeit to a lesser degree [3]. The processes exhibiting AOP characteristics include photocatalysis, piezocatalysis, pyrocatalysis, or a combination of any of these processes.

In the context of water-cleaning applications, ferroelectric materials have emerged as a boon to catalysis processes [4,5]. Ferroelectric materials are a class of materials that exhibit a unique property known as ferroelectricity whereby the spontaneous electric polarization can be reversed by applying an external electric field [6]. Due to this versatility of ferroelectric materials, they are utilized in diverse fields such as energy storage, energy-harvesting systems, actuators, sensors, ultrasound transducers, energy conversion, human health treatment, neuromorphic devices, etc. [7,8,9,10,11,12,13,14]. 

Focusing on water remediation, the advantageous effect of ferroelectric materials in photocatalysis stems from the presence of a built-in electric field, which effectively facilitates the separation of photogenerated charge carriers [15]. Further, the process involving the generation of polarization induced by mechanical stress, commonly known as piezoelectricity, has been extensively harnessed as a driving force for the catalytic degradation of organic pollutants, even in the absence of light. In recent times, a novel approach to enhancing the degradation efficiency of photocatalysis has emerged, involving the modulation of polarization in ferroelectric materials [15]. This method has shown great promise in boosting photocatalytic performance significantly. The combination of piezoelectricity and photocatalysis holds great potential for efficiently breaking down organic pollutants and holds promise for various environmental remediation applications. It is widely recognized that all ferroelectric materials exhibit pyroelectric properties, enabling them to convert thermal energy into electricity when subjected to temperature fluctuations [16]. Furthermore, the phenomenon of the synergistic effect of possible catalysis processes has been acknowledged as a highly effective method to improve catalytic performance. Additionally, by utilizing ferroelectric switching, it becomes possible to control the chemical properties of catalyst surfaces, leading to a substantial improvement in catalytic efficiency. This opens up new avenues for developing high-performance catalysts by incorporating ferroelectric materials into their design [17].

BaTiO_3_ (BT) has received the most attention among conventional ferroelectric materials due to its outstanding dielectric, ferroelectric, optical, piezoelectric, and other important characteristics [4,18,19,20,21,22,23,24]. It is well known that the crystal structure of BT stabilizes, depending on temperature, into rhombohedral *R3m*, orthorhombic *Amm2*, tetragonal *P4mm*, and cubic *Pm3m* phases [25]. Additionally, by adding appropriate impurities or substituting certain crystalline phases in BT, these characteristics and/or crystalline phases can be adjusted and optimized. Ferroelectric ceramics are hardly used in pure chemical form; instead, doping is always used to modify the characteristics for a given application. Donor doping to obtain high piezoelectric coefficients and acceptor doping to achieve low dielectric losses are two examples of how different dopings typically have distinct effects on BT ceramics [26]. Further, equivalent doping at the A-site and B-site is also another approach for tailoring the properties of BT ceramics considering the morphotropic phase boundary (MPB). The simultaneous doping of Ca and Zr in a BT ceramic, making it a (Ba,Ca,Zr,Ti)O_3_ composition, is one of the most employed compositions for various applications. There are many studies on the (Ba,Ca,Zr,Ti)O_3_ composition related to dielectric behavior, caloric effects, actuation, sensing, and pyroelectric behavior.

BCZTO stands out as a unique composition within the (Ba, Ca, Zr, Ti)O_3_ system, offering intriguing possibilities for exploring its potential as a multicatalyst in environmental remediation through various catalysis processes. These processes encompass photocatalysis, piezocatalysis, piezo-photocatalysis, and pyrocatalysis. Despite the limited existing research on BCZTO as a catalyst for environmental remediation, understanding its fundamental catalytic mechanisms and adaptability is crucial. In this review, we focus on delving into the multicatalytic applications of BCZTO ferroelectric materials. Specifically, we thoroughly investigate their catalytic performance in key reactions such as photocatalysis, piezocatalysis, and pyrocatalysis. Through a comprehensive analysis of the underlying mechanisms and performance metrics, we emphasize the versatility of BCZTO ferroelectric material as a highly promising multicatalyst.

## 2. Ferroelectric Materials 

### 2.1. Fundamentals of Ferroelectric Materials

**Figure 1:**
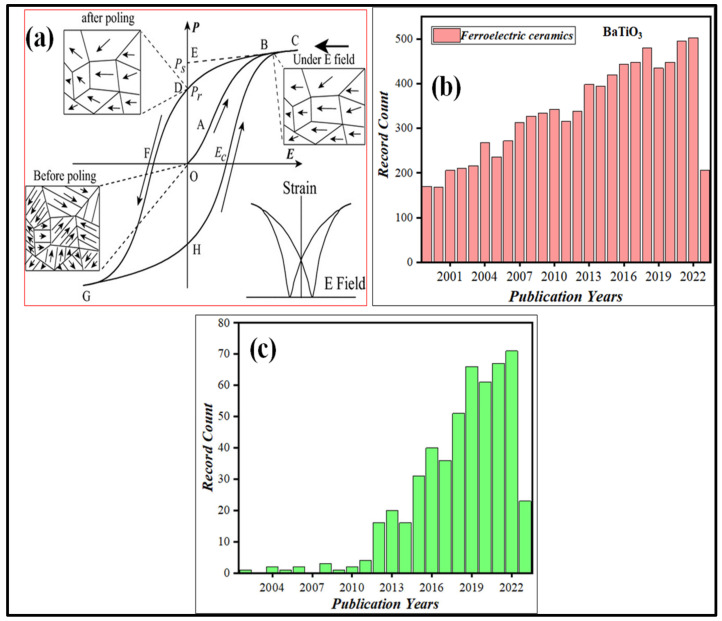
(**a**) Ideal hysteresis loop of ferroelectric ceramics with electric field–strain curve. Reproduced with permission from Shujun Zhang et al., Journal of American Ceramic Society; published by John Wiley and Sons, 2013 [6]. Number of published articles since 2000 on (**b**) BaTiO_3_ ceramics and (**c**) BCZTO ceramics for various applications (data taken from Web of Science, searched as “BaTiO_3_ ceramic” and “Ba_0.85_Ca_0.15_Ti_0.9_Zr_0.1_O_3_ ceramic”, dated 26 June 2023).

There have been numerous developmental milestones since the discovery of ferroelectricity in Rochelle salt about a century ago, which have all been thoroughly examined by Kanzig, Cross, and Newnham, as well as Fousek, and Haertling [27,28,29,30,31,32,33]. The most prevalent characteristic of ferroic materials is the emergence of a domain structure through spontaneously breaking prototypal symmetry, manifesting as hysteresis loops with corresponding conjugate fields. Every ferroelectric material has its distinct hysteresis loop, like a fingerprint. Hysteresis loops allow for the immediate identification of ferroelectricity. Figure 1a shows the hysteresis loop of a typical ferroelectric material. Initially, the domain directions are randomly distributed to result in zero net macroscopic polarization. The polycrystalline ferroelectric ceramic may be brought into a polar state when the external field is greater than the coercive field (*E_c_*).

An increase in the electric field strength causes gradual macroscopic polarisation, as demonstrated in Figure 1a. The dramatic polarization change near *E_c_* is primarily due to polarization reversal (domain flipping), but at the high-field end, the polarization is saturated and the material behaves as a linear dielectric [6,34]. There is a back-switch in some domains as the electric field intensity decreases, but at zero field the network polarization is non-zero, resulting in remnant polarization (*P_r_*). Since ferroelectrics typically have ferroelastic regions, except for LiNbO_3_, which only contains 180° of ferroelectric regions, the simultaneous induction of spontaneous strain by an external electric field is also possible [6]. Therefore, a strain–electric field curve, or “butterfly,” can be seen if the strain is measured along with the polarization. The observed hysteresis loops are symmetric in an ideal ferroelectric system, resulting in an equal positive and negative *E_c_* and *P_r_*. Ferroelectric materials, due to their inherent property of spontaneous polarization, have significant applications in various fields such as energy harvesting, energy storage, bio-materials, environmental remediation, and the electronics industry. Several ferroelectric ceramics have been identified for these diverse applications, including BaTiO_3_ (BT), Pb(Zr,Ti)O_3_ (PZT), (K,Na)NbO_3_ (KNN), LiNbO_3_ (LN), BiFeO_3_, and Bi_4_Ti_3_O_12_, among others [35,36,37,38,39,40,41,42,43]. 

As discussed earlier, the BT ceramic is one of the most studied ferroelectric materials. Figure 1b shows the data on the studied BT ceramics from 2000 to now for various applications. Doping is one way of enhancing the physical and chemical properties of BT ceramics. In the later section, we will review one such composition, 0.5(Ba_0.7_Ca_0.3_)TiO_3_-0.5Ba(Zr_0.2_Ti_0.8_)O_3_ or Ba_0.85_Ca_0.15_Ti_0.9_Zr_0.1_O_3_ (BCZTO), where both the Ca and Zr elements are doped simultaneously for multicatalytic activities. Figure 1c shows the data on the studied BCZTO ceramics from 2000 to now.

### 2.2. Ba_0.85_Ca_0.15_Ti_0.9_Zr_0.1_O_3_ (BCZTO) Ceramics

**Figure 2:**
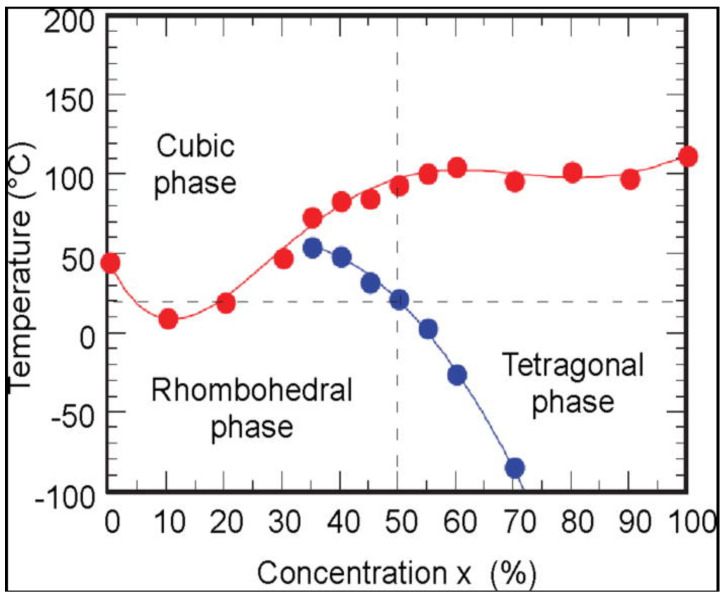
Phase diagram for BCT–BZT system. Reproduced with permission from Turygin, A.P. et al., Journal of Applied Physics; published by AIP Publishing, 2015 [46].

BCZTO has shown great potential in various emerging energy-harvesting fields due to its numerous advantages. BCZTO is a noteworthy material due to its lead-free composition, which renders it environmentally sustainable [44]. It demonstrates a noteworthy Curie temperature (*T_c_*) of approximately 85 °C, coupled with compelling electrical characteristics [45]. Recent studies have indicated noteworthy advancements in the field of piezoelectric and coupling coefficients of BCZTO. Piezoelectric coefficients (*d*_33_) greater than or equal to 630 pC/N and high coupling coefficients (*k_p_*) of 0.56 have been observed, which are comparable to those found in the Pb-based materials currently in use [45]. The exceptional values exhibited by BCZTO indicate its potential as a superior alternative. The tunable, electric-field-dependent dielectric properties of BCZTO render it suitable for a wide range of applications. In addition, it has a low dielectric loss of 0.01, which suggests minimal energy dissipation. The favorable amalgamation of characteristics can be ascribed to the concurrent presence of two distinct phases, namely rhombohedral (R) and tetragonal (T), along with an intervening orthorhombic phase (Figure 2) [46].

This distinctive arrangement significantly reduces the energy barrier for switching, thereby enhancing the outstanding properties of BCZTO. Considerable research has been carried out to investigate the impact of the composition and MPB on the ferroelectric characteristics of BCZTO [47]. The high piezoelectric properties are achieved at the tricritical point (TCP), which is defined by the simultaneous presence of R, T, and cubic (C) phases at a single point. At the TCP, the energy barrier for the rotation of polarization between the T and R states is low or at its minimum (soft), allowing the direction of polarization to be easily influenced by external pressure or electric fields [48]. This promotes a high level of piezoelectricity and permittivity. Furthermore, it has been reported that lead-free piezomaterial systems contain a triple point where the C–R–T phases coexist. The aforementioned studies have yielded comprehensive insights into the factors that impact the ferroelectric behavior of BCZTO, thereby laying the foundation for further advancements in this promising material.

**Figure 3:**
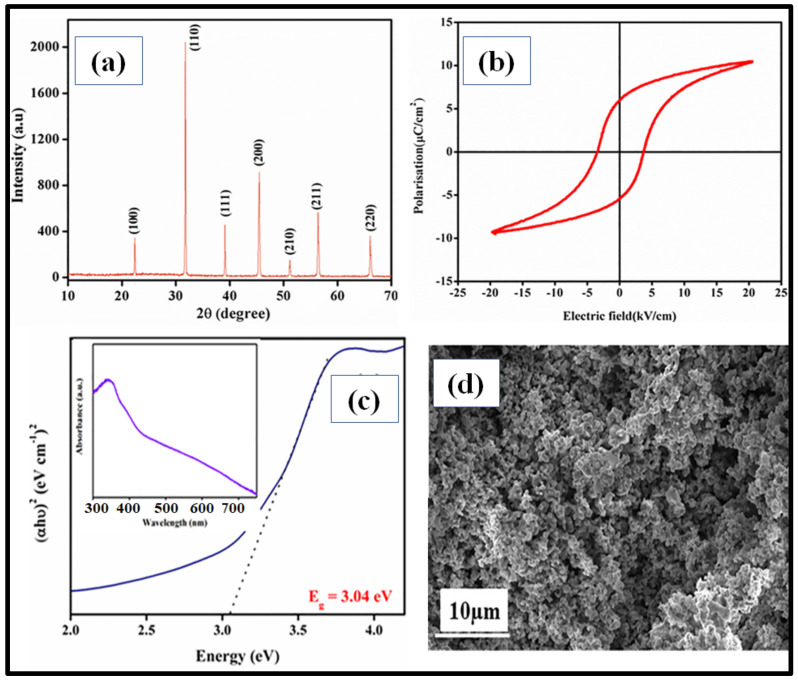
BCZTO ceramics. Reproduced with permission from Moolchand Sharma et al., Journal of Applied Physics; published by AIP Publishing, 2020 [49]. (**a**) X-ray diffraction pattern (XRD); (**b**) P–E loop at room temperature; (**c**) absorbance spectrum (inset) showing Tauc’s plot revealing energy band gap; (**d**) surface morphology of synthesized BCZTO ceramic via scanning electron microscopy (SEM).

Figure 3a shows the XRD pattern of the BCZTO ceramic (pellet) synthesized via a solid-state reaction method (precursors calcined at 1190 °C for 6 h, pellet sintered at 1400 °C for 4 h) where the precursors were manually milled and no traces of the impurity were detected. Figure 3b shows the ferroelectric hysteresis loop for the BCZTO ceramic, taken at a temperature of 303 K and a frequency of 50 Hz. The curve clearly shows that as the applied electric field is increased, the polarization grows until it reaches a saturation point. The fact that some residual polarization persists in the ceramics after the removal of the external electric field proves that the BCZTO ceramic is a ferroelectric material. Figure 3c demonstrates the absorbance spectra of the BCZTO ceramic, and Tauc’s plot shows its energy band gap to be ~3.04 eV. Further, Figure 3d represents the SEM images of the synthesized BCZTO ceramic showing the surface morphology in an irregular shape obtained by a solid-state reaction method.

## 3. Multicatalytic Ability of Ferroelectric Materials

There are various methods and catalyst materials used for water-cleaning applications, among which titanium dioxide (TiO_2_) stands out due to its excellent electrochemical properties and stability [50]. However, its efficiency is hampered by significant interior and surface defects, leading to the unwanted recombination of photogenerated electrons and holes during photocatalysis, resulting in reduced energy conversion efficiency [50]. To address these limitations, different techniques have been employed to enhance the charge separation and transport properties in oxide materials, such as surface functionalization, heterojunction formation, and defect adjustment [51,52,53]. Indeed, carbonaceous materials have also been explored as catalyst enhancers for photocatalytic activities. These materials, including activated carbon, carbon nanotubes, and graphene, possess properties that can improve the performance of photocatalysts. However, as mentioned before, they are expensive to produce and involve complex fabrication procedures, which can limit their widespread application in certain contexts [54].

Recently, a novel strategy has emerged involving the use of ferroelectric polarization to control electrochemical processes and modulate the interfacial electronic structure, which has gained considerable attention in photocatalysis [55]. This newfound focus on utilizing ferroelectric and piezoelectric materials in photocatalysis has led to improved charge separation. By combining piezoelectric materials with ferroelectric properties alongside other photocatalysts, the inherent electric field near the piezoelectric material plays a crucial role in facilitating the separation of charges. As a result, the use of ferroelectric materials in water remediation has increased over time. Moreover, ferroelectric materials, being both piezoelectric and pyroelectric, offer the advantage of mechanical-stress-induced localized polarization charges, which can effectively control the carrier generation, separation, transport, and recombination processes [55]. Consequently, ferroelectric materials are employed in photo/pyro/piezo/piezo-photocatalysis processes for pollutant removal applications. In the subsequent sections, the specific mechanisms involved in these catalysis processes are elaborated, and later the multicatalytic abilities of the BCZTO ceramic are analyzed. 

### 3.1. Photocatalysis Process

In comparison to conventional adsorbents such as active charcoal, metal–organic frameworks, and silica gel, the photocatalytic degradation process offers greater reliability. This is because the recycling of these adsorbents requires harsh conditions to restore their adsorptive capacity, often involving non-environmentally friendly chemicals and solvents that are expensive and lead to secondary pollution [56]. The photocatalytic degradation technique employs an advanced oxidation process. During photocatalysis, highly reactive species are generated, which rapidly attack the organic pollutant molecules and oxidize them in a shorter timeframe. This approach proves more efficient and environmentally friendly, making it a preferred choice over traditional adsorbents.

The process makes use of the energy of illuminating light (visible/UV/solar), whereby the catalyst absorbs energy from the light corresponding to the energy band gap of a catalyst. Indeed, this is the fundamental step involved in all photocatalytic processes. When light with enough energy strikes a semiconductor material, it triggers the excitation of electrons (*e^−^*) from the valence band (VB) to the conduction band (CB), creating positively charged holes (*h^+^*) in the valence band. This photoexcitation process is the starting point for various photocatalytic reactions [37,57]. The photogenerated pairs (*e^−^/h^+^*) have the potential to move to the catalyst surface and take part in photocatalytic surface redox reactions before undergoing any recombination process. If the reduction potential of these electrons and holes meets the application requirements, then they can be utilized for diverse photocatalytic reactions.

Based upon the capability of the band energy of the catalyst, the *e^−^* in the conduction band acts as an oxidizing agent causing adsorbed oxygen (O_2_) to produce superoxide (**^.^**O_2_*^−^*) radicals, whereas the *h^+^* in the valence band acts as a strong reducing agent responsible for the production of OH from H_2_O [58]. 

Due to their high reactivity, these active species efficiently break down pollutant molecules into smaller fragments, ultimately leading to the complete mineralization of pollutants over time. The degradation reaction takes place on the catalyst’s surface, where the degraded products are released (desorbed) while new pollutant molecules are adsorbed. This cyclic process ensures the completion of the degradation [59]. Furthermore, to achieve high photocatalytic activity, it is essential to inhibit the recombination of photogenerated charges, thus enabling the efficient generation of these active species. 

Utilizing ferroelectric semiconductors offers a viable strategy to enhance photocatalytic activity by introducing a permanent internal polarization. This polarization plays a crucial role in efficiently separating photoexcited carriers. Ferroelectric materials possess distinctive photochemical properties associated with the internal dipole of the material, which stems from the non-centrosymmetric nature of their crystal structure [60]. These unique characteristics contribute to their enhanced performance in photocatalysis. Thus, being a reliable and effortless process, the photocatalysis process finds its applicability in the treatment of wastewater, air purification, carbon dioxide (CO_2_) reduction, hydrogen evolution, self-cleaning surfaces, detoxification, and bacterial disinfection [61,62,63].

**Figure 4:**
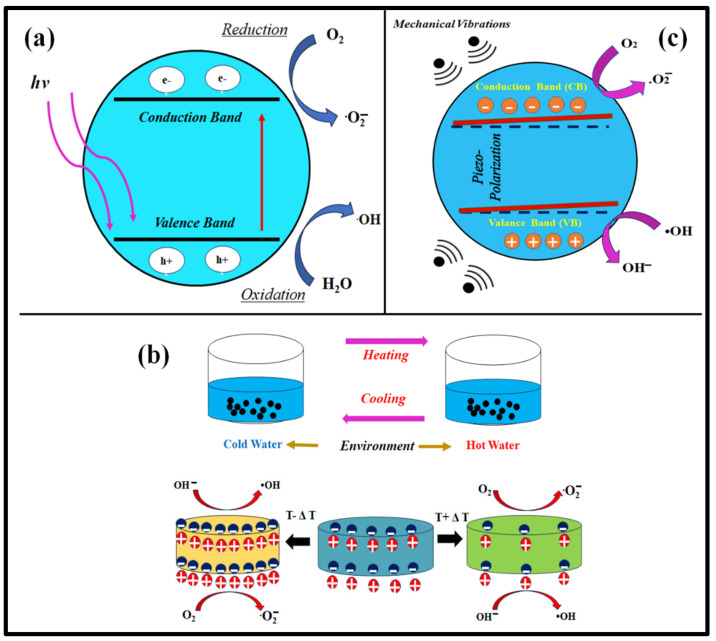
Schematic representation of (**a**) photocatalysis, (**b**) pyrocatalysis, and (**c**) piezocatalysis process.

The schematic representation of the photocatalysis process is shown in Figure 4a.

### 3.2. Pyrocatalysis Process

The pyroelectric effect is an intriguing phenomenon, which enables catalytic operations to utilize waste heat energy. It has been well-established for a very long time that some crystals respond to temperature variations by forming a spontaneous polarization on their surfaces [64,65]. For many years, pyroelectric materials have been used in conventional applications including temperature sensing and imaging [66]. Microscopic pyroelectric materials have been researched recently for more modern uses, such as surface chemical catalytic processes. For processing redox reactions, pyroelectrics utilize the energy from heat effects due to temperature differences. The crucial factors responsible for the pyrocatalysis process are the thickness of the catalyst, rate of achieving the temperature difference, and working temperature <*T_c_*.

Ferroelectric materials typically exhibit their most significant alteration in polarization and peak pyroelectric characteristics in the vicinity of *T_c_* [67]. As a result, one can anticipate a notably improved catalytic performance in the vicinity of *T_c_*. To prove such a phenomenon, a lead-free BaTi_0.89_Sn_0.11_O_3_ ceramic (*T_c_* ~ 40 °C) was utilized for degrading Rhodamine B (RB) dye in the hot–cold fluctuation range of 25–45 °C (across *T_c_*) and 5–25 °C (below *T_c_*), where the degradation of RB dye was relatively high in the vicinity of its *T_c_* compared to the lowered operating fluctuation temperature [68]. As a fact, when a significant temperature difference is applied to a pyrocatalyst, the charges undergo separation, leading them to engage in redox reactions. Further, the improved degradation of dye can be attributed to the development of an internal electric field that enhances the separation of positive and negative carriers, thereby facilitating their migration at an increased rate. The efficiency of photocatalytic activity relies on the efficient separation of photo-induced electron–hole pairs for electrochemical redox reactions. This separation process can be enhanced through various methods, including the creation of heterojunctions, the introduction of an additional electric field, and the deposition of noble metals [69]. Coating noble metals on the surface of pyroelectric nanomaterials is expected to enhance the efficiency of pyrocatalytic dye decomposition, similar to the photocatalysis process. One of the studies showed similar results where Ba_0.7_Sr_0.3_TiO_3_@Ag pyroelectric nanoparticles caused the enhanced decomposition of RB dye compared to bare Ba_0.7_Sr_0.3_TiO_3_ pyroelectric nanoparticles [69]. The improved efficiency of pyrocatalytic dye decomposition, resulting from coating silver on the Ba_0.7_Sr_0.3_TiO_3_ surface, can be attributed to the impediment of recombination between pyroelectrically induced positive and negative electric charges. The generation of a heterojunction significantly enhances the pyrocatalytic performance. The BiFeO_3_/g-C_3_N_4_ heterostructure for pyrocatalysis process was investigated in one such study for the validation of such a statement, where compared to pure BiFeO_3_, the BiFeO_3_/g-C_3_N_4_ heterostructure showed improved pyrocatalytic activity [70]. This remarkable enhancement can be attributed to the creation of an internal electric field, which promotes the efficient separation of positive and negative carriers and accelerates their migration. A schematic representation of the pyrocatalysis process is shown in Figure 4b.

### 3.3. Piezocatalysis Process

It is well known that ferroelectric materials are also piezoelectric, thereby providing an immense space for ferroelectric materials to be utilized in the piezocatalysis process. Piezocatalysis is a recently developed, promising technology exhibiting amazing performance in clean energy conversion and water treatment by gathering mechanical energy such as vibration, friction, natural wind, and tides [71]. In 2010, Hong et al. published the first report on the application of piezoelectric materials in chemical catalysis [72]. BaTiO_3_ microdendrites were used for water splitting, which involves the production of hydrogen and oxygen by splitting water. Further, Hong et al. conducted a study where they reported on the degradation of the organic dye acid orange (AO7) using piezoelectric BaTiO_3_ microdendrites [73]. You et al. demonstrated the piezocatalytic activity of ferroelectric BiFeO_3_ for the degradation of RB dye [74]. Since then, ferroelectric materials for water applications have evolved with increasing scientific knowledge [15,75,76,77,78,79]. Ferroelectric materials are versatile, as they not only enable water splitting and dye degradation but also possess bacterial disinfection properties by disinfecting bacteria such as Gram-positive bacteria (*S. aureus*) and Gram-negative bacteria (*E. coli*) [80]. 

Upon the application of mechanical stress, the charges are separated creating an electric field, and as a result, local polarization within the catalyst takes place. There are two theories reported in the literature for piezocatalysis, i.e., the energy band gap theory and screening effect theory [81].

#### 3.3.1. Energy Band Gap Theory

This theory says that the piezocatalyst band configuration and electronic states are the primary factors that regulate catalytic activity. In contrast to a perfect insulator, where no mobile charges can move in response to the piezoelectric potential field, the band level tilts linearly across the strained zone, and the oriented accumulation of internal mobile charges can screen the band tilting of piezoelectric semiconductors [82].

#### 3.3.2. Screening Effect Theory

In contrast to the energy band theory, the mechanism of the screening charge effect places more emphasis on the piezopotential’s dominant roles and its associated surface screening behavior in piezoelectric materials. The magnitude of the piezopotential should exactly match the required redox level to be compatible with electrocatalysis and to determine the ability of the piezocatalyst to accomplish a certain chemical reaction. More crucially, the polarization-induced surface screening behavior regulates the piezocatalytic process.

In piezocatalysis, the application of external stresses, especially through an acoustic field, induces bulk polarization in the piezocatalyst. This polarization leads to deformation and charge separation within the piezocatalyst, creating a “top-to-bottom” potential difference. This difference enables redox reactions to take place at different points on the piezocatalyst surface, ultimately leading to the degradation of organic pollutants.

### 3.4. Multicatalytic Capability of BCZTO

The phenomenon of dipole randomization after sintering, which is crucial for producing ceramics in bulk form, can hinder catalytic processes. However, this limitation can be overcome by efficiently orienting the dipoles through the application of an electric field. This controlled orientation of dipoles leads to a significant enhancement and improvement in the efficiency of the catalysis process. The ferroelectric BCZTO ceramic exemplifies such a mechanism, where the effective orientation of dipoles contributes to its catalytic prowess.

**Figure 5:**
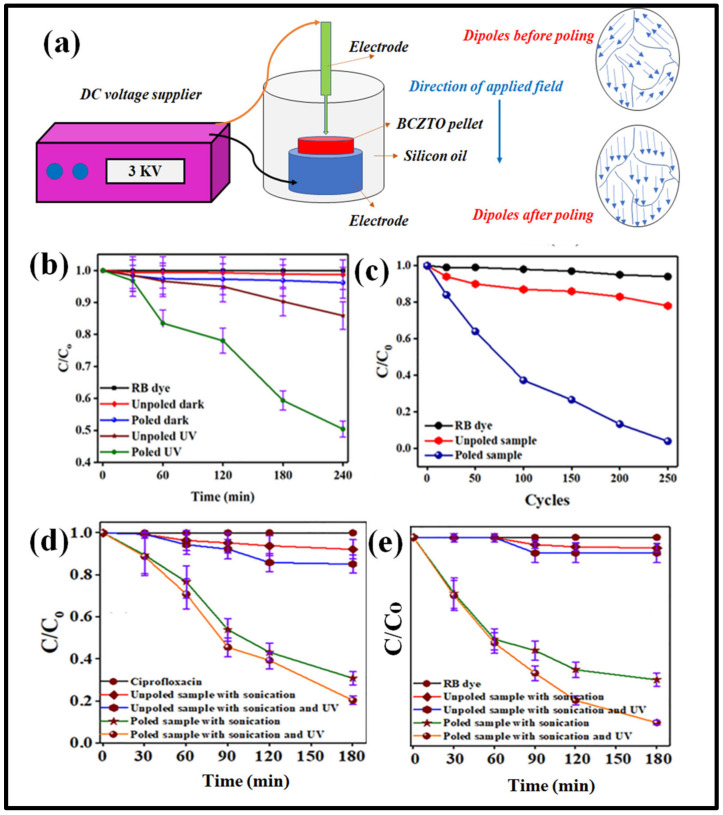
(**a**) Illustration of conventional DC poling for ferroelectric material; the effect of poling using BCZTO ceramics. Reproduced with permission from Moolchand Sharma et al., Journal of Applied Physics; published by AIP Publishing, 2020 [49]. (**b**) Photocatalysis process (UV source) for degrading ~10 mg/L concentrated RB dye (20 mL); (**c**) pyrocatalysis process (in range of 2 °C and 40 °C) for degrading ~10 mg/L concentrated RB dye (20 mL); (**d**,**e**) piezo-photocatalysis process (UV source, ultrasonicator (40 kHz, 70 W)) for degrading ~10 mg/L concentrated ciprofloxacin (20 mL) and ~10 mg/L concentrated RB dye (20 mL), respectively.

In a particular study, the multicatalytic capability of a BCZTO ceramic was investigated, revealing that the ceramic exhibited a relatively low degradation rate (~35% in 210 min) of a concentrated Methylene Blue (MB) dye (20 mL, ~5mg/L) when exposed to visible light from a 15 W source [83]. This reduced photocatalytic activity was attributed to the energy band gap of the BCZTO ceramic, which naturally corresponded to ultraviolet (UV) light energy, making it less responsive to the energy of visible light. Consequently, the ceramic pellet did not generate enough electron–hole pairs necessary for efficient MB dye degradation. Further in the study, the piezocatalytic and pyrocatalytic properties of the BCZTO ceramic were also studied for MB dye degradation. Through a conventional poling method (3 kV/mm in a silicon oil bath), the BCZTO pellet’s catalytic efficiency improved significantly for all processes compared to the unpoled counterpart. Even under UV irradiation for photocatalysis, the poled BCZTO pellet demonstrated better performance than the unpoled one. The poled BCZTO pellet exhibited promising piezocatalytic activity, degrading approximately 94% of MB dye within 150 min. During pyrocatalysis, with around 200 cycles and a temperature maintained between 10–45 °C, the poled BCZTO ceramic displayed an excellent pyroelectric effect, achieving approximately 92% degradation of MB dye. In reality, domains within the materials are arbitrarily orientated as soon as the polycrystalline ferroelectric material is synthesized by the sintering process; ceramic initially lacks piezoelectric characteristics [27]. However, when the ferroelectric materials move through a strong electric field, the randomly oriented dipoles are aligned in the direction of the applied electric field; thus, charge separation during mechanical vibration becomes systematic and easy. This alignment resulted in a delay in the recombination rate within the catalyst, leading to higher multicatalytic efficiency. Overall, the study demonstrated that the application of an electric field through poling significantly improved the catalytic efficiency of the BCZTO ceramic, making it a promising candidate for various multicatalytic processes. Figure 5a shows the illustration of poling the ferroelectric ceramic via a conventional DC poling setup. 

In a different study, a BCZTO ceramic was employed as a catalyst with a UV light source (365 nm wavelength) for the photocatalysis process, targeting the degradation of RB dye and ciprofloxacin (20 mL and ~10 mg/L concentrated) as representative pollutants [49]. The investigation focused on the impact of conventional poling on the BCZTO ceramic pellet. The results showed that the degradation of RB dye through photocatalysis using UV light was approximately 3.4-fold higher with the poled BCZTO pellet compared to the unpoled BCZTO ceramic pellet. This significant enhancement in photocatalytic efficiency demonstrated the positive effect of poling on the catalytic performance of the BCZTO ceramic. The coupling of piezocatalytic and photocatalytic properties within the ferroelectric material resulted in the increased efficiency of the photocatalytic process. 

Similar findings were observed with 0.5Ba(Zr_0.2_Ti_0.8_)O_3_–0.5(Ba_0.7_Sr_0.3_)TiO_3_ (BST–BZT) composition where the poled sample showed enhanced catalytic performance compared to unpoled sample, with delayed recombination being one of the factors for the improved performance [4].

Notably, the degradation of organic pollutants was significantly higher in the combined piezo-photocatalysis process compared to individual piezocatalysis and photocatalysis for both unpoled and poled samples, as shown in Figure 5d,e.

The synergistic piezocatalysis and photocatalysis effect is the result of combining the piezocatalytic and photocatalytic processes. When piezoelectric polarization is induced by the ultrasonic wave in the ferroelectric material, electrons and holes are polarized along its spontaneous polarization axis. This creates a p–n junction with a steeper band alignment compared to those activated solely by light irradiation in the photocatalytic process. If polarization is oriented in one direction, then electrons will indeed move in the opposite direction, whereas the holes will follow the opposite path of electrons. These movements of electrons and holes in response to the piezoelectric polarization demonstrate the role of mechanical force in influencing charge transport and separation within the material. This phenomenon is crucial in the synergistic piezo- and photocatalysis effect, as it facilitates enhanced charge separation and contributes to the improved catalytic performance of the material [84].

In line with the observations for the pharmaceutical drug, a similar trend was noted in the degradation of organic pollutants using poled BCZTO ceramic pellets. The effect of poling on the BCZTO ceramic was also observed in the pyrocatalysis process, where a poled BCZTO ceramic showed ~97% of RB dye degradation when the temperature conditions were set between 2 and 40 °C with pyrocatalysis running for 250 cycles, as shown in Figure 5c. To support such a theory of enhanced pyrocatalytic activity through electric poling for dye degradation applications, in one of the studies, BaTiO_3_ crystal was electrically poled under different fields, where the maximum improved performance for the poled BaTiO_3_ crystal was 7-fold greater than the unpoled BaTiO_3_ crystal, which was due the increased number of participating charges in the redox reaction [85].

Moreover, BCZTO ceramics exhibited excellent reusability as a catalyst, showing negligible performance changes even after five cycles of usage in the piezo-photocatalysis process. The effectiveness of poling in the BCZTO ceramic was evident across various processes, including photocatalysis, pyrocatalysis, and piezo-photocatalysis, as depicted in Figure 5b–e. Furthermore, the versatility of BCZTO pellets was assessed in bacterial disinfection experiments. Poled BCZTO samples proved to be more effective in killing both Gram-positive bacteria (*S. aureus*) and Gram-negative bacteria (*E. coli*) during the piezocatalysis process compared to unpoled BCZTO ceramics. Generally, poled BCZTO can cause water molecules to form ROS, which have a degenerative effect on bacterial cells. Highly reactive species (ROS) damage bacterial cells by destroying everything that attacks bacterial cells from lipids in the cell membrane to nucleotide bases and sugar in DNA [86]. 

These findings collectively demonstrate the promising potential of the BCZTO ceramic as a multifunctional material with enhanced catalytic properties due to thr poling effect, making it a valuable candidate for various applications in water purification, environmental remediation, and bacterial disinfection processes.

Organic pollutant degradation using visible light via the photocatalysis process has become a focus of interest for future prospects. The doping of a transition metal into the parent compound results in reducing the energy band gap of the synthesized ceramics. By incorporating Iron (Fe) into the parent compound, the energy band gap between the conduction and valence bands decreases, enabling the ceramics to absorb visible light. This Fe^+3^ doping in BCZTO enhances visible light photocatalytic activity by creating defects such as vacancies and narrowing the effective band gap. Similar photocatalysis via visible light has been reported in the literature with Fe doping in BaTiO_3_ [18]. However, tetragonality in BCZTO diminishes with Fe substitution. In addition, the ferro-para phase transition was diffused following Fe doping, indicating that the long-range interactions between ferroelectric domains and defects caused by Fe were broken. Additionally, a few reports are suggesting that adding Fe could improve the BCZTO electrocaloric and other electrical properties [87,88]. As a fact that doping is beneficial in decreasing the energy band gap of BCZTO, it was introduced in BCZTO as Ba_0.85_Ca_0.15_(Ti_0.9_Zr_0.1_)_1−x_Fe_x_O_3._

**Figure 6:**
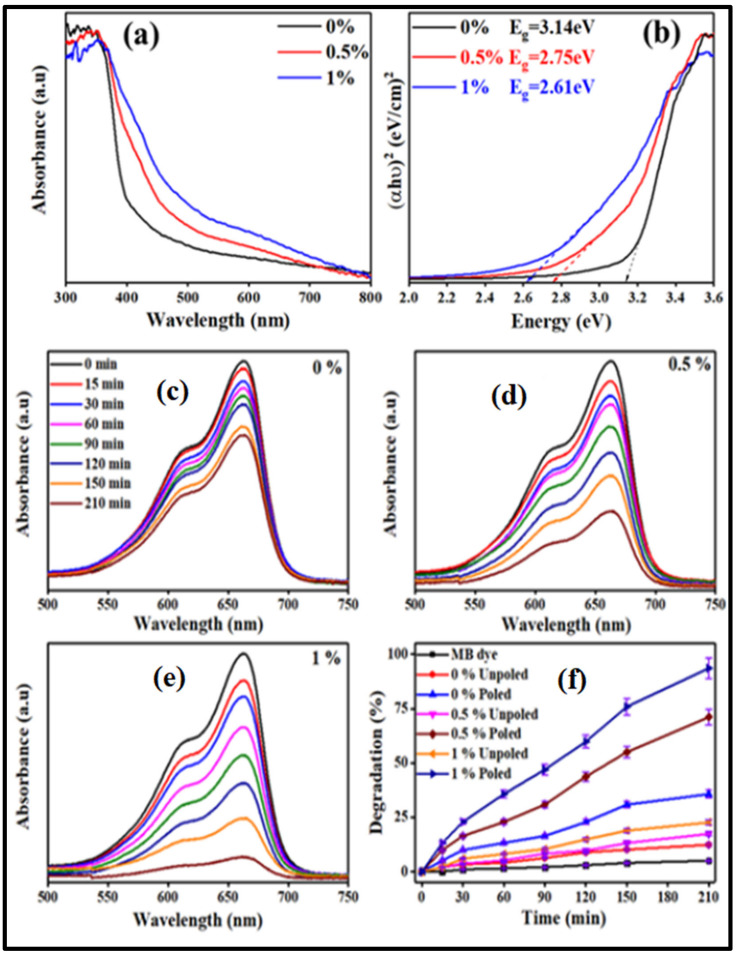
(**a**) Absorbance spectra of 0, 0.5, and 1% Fe-doped BCZTO ceramic (Ba_0.85_Ca_0.15_(Ti_0.9_Zr_0.1_)_1-x_Fe_x_O_3_ ceramics) in the wavelength range of 300–800 nm; (**b**) Tauc’s plot showing energy band gap of 0, 0.5, and 1% Fe-doped BCZTO ceramic; (**c**–**f**) photocatalytic performance of Ba_0.85_Ca_0.15_(Ti_0.9_Zr_0.1_)_1-x_Fe_x_O_3_ composition for degrading ~5 mg/L concentrated MB dye. Reproduced with permission from Moolchand Sharma et al., Journal of American Ceramic Society; published by John Wiley and Sons, 2020 [83].

With the inclusion of Fe in the BCZTO lattice, the energy band gap of the composition decreased drastically, and the band gap of the synthesized samples containing 0%, 0.5%, and 1% Fe was determined to be around 3.14, 2.75, and 2.61 eV as shown in Figure 6a,b. When the Fe content of BCZTO ceramics rose, the band gap shifted from the UV to the visible region. The electronic states of the highest occupied molecular orbital (HOMO) and the lowest unoccupied molecular orbital (LUMO), which were created by Fe substitution, were used to explain the influence of Fe on the optical band gap (for BCZTO) in the stated literature [89]. Near the top of the valence band and bottom of the energy spectrum, oxygen vacancies produced extra energy states. This resulted in enhanced photocatalytic activity for MB dye with an increase in Fe doping in the BCZTO ceramic, as shown in Figure 6c–f. However, pyrocatalysis and piezocatalysis activity decreased with an increase in Fe doping. The reason could be due to the decrease in the piezoelectric coefficient (*d*_33_) with an increase in Fe content. 

It has been reported in the past that adding noble metals (Ag, Au, etc.) to ferroelectric materials can increase the activity in piezocatalysis and cause visible light photocatalysis [90,91]. Carrying forward this approach of enhanced activity in photocatalysis and piezocatalysis processes for degrading organic contaminants, Moolchand et al. loaded Ag onto a BCZTO ceramic and investigated its photo/piezocatalysis performance [92]. The loading of the Ag element onto the BCZTO ceramic was performed by dissolving as-prepared BCZTO ceramic powder and AgNO_3_ in ethylene glycol solution. The solution was then stirred for around 2 h, after which the solution was repeatedly washed with water and acetone with further exposure to the drying temperature [93]. RB dye was nearly destroyed completely in an aqueous solution in just 40 min utilizing the Ag-loaded BCZTO sample, demonstrating the promising photocatalytic activity of the sample. Further, an Ag-loaded BCZTO sample was used in the piezocatalysis method to demineralize around 95% of the RB dye, demonstrating the promising piezocatalytic potential of BCZTO ceramic. The improved catalytic characteristics of the BCZTO ceramic arise from the occurrence of surface plasmonic resonance (SPR). One of the manifestations of SPR was the emergence of a hump in the absorbance spectrum within the visible range when Ag was incorporated into the BCZTO ceramic [94]. This hump in the visible range of the absorbance spectrum corresponds to the energy of falling visible light, while the energy band gap of the BCZTO and Ag-loaded BCZTO ceramic remained the same. A similar observation of a developed hump in the visible range of the absorbance spectrum due to SPR is reported in the literature [95]. 

The recovery of catalysts after usage is a challenging task, but one way to facilitate this is by embedding the catalyst in a suitable matrix. This approach becomes even more advantageous when the matrix itself contributes to the catalysis process. One of the novel techniques involves the precipitation of a ceramic phase within a glass matrix, which has been employed as a successful catalyst [37,96]. However, glass–ceramics are inherently brittle, limiting their effectiveness in certain catalysis processes that require flexibility. To address this limitation and combine catalytic properties with flexibility, polymers are considered potential matrices for catalysts. Their flexible characteristics make them a promising option for catalysis applications.

**Figure 7:**
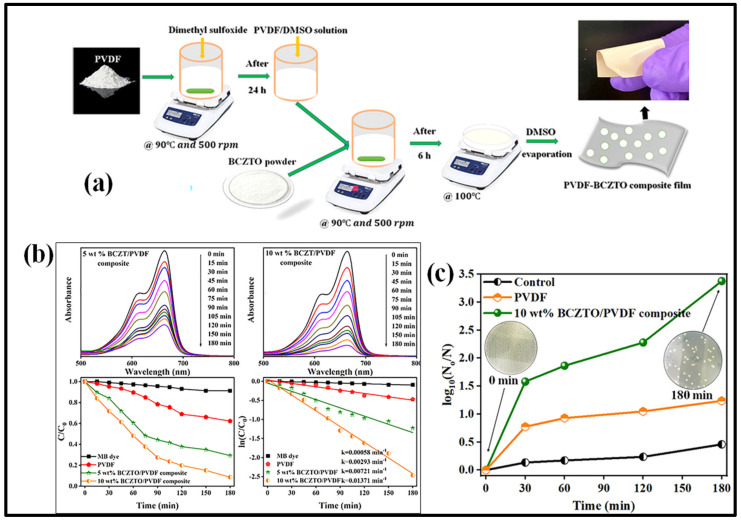
(**a**) Schematic representation of BCZTO/PVDF film synthesis, (**b**) degradation results of MB dye via piezocatalysis using BCZTO/PVDF film, and (**c**) bacterial disinfection by 10 wt.% BCZTO ceramic/PVDF film through piezocatalysis process. Reproduced with permission from Moolchand Sharma et al., Journal of Applied Physics; published by AIP Publishing, 2021 [97].

Ferroelectric BCZTO ceramic powder at 5 and 10 wt.% were immobilized in a matrix of PVDF by Moolchand et al., making a flexible and stable composite film [97]. Polyvinylidene difluoride (PVDF) and its derivatives has piezoelectric characteristics due to which it is used as flexible catalyst [98]. Figure 7a shows the synthesis route of the PVDF–BCZTO ceramic composite. It is obvious that 10 wt.% would have shown excellent piezocatalysis catalysis due to the high content of BCZTO ceramic in PVDF. The 10 wt.% BCZTO ceramic–PVDF composite film degraded ~91% MB, ~86% RB, and ~90% Methylene Orange (MO) organic dyes via the piezocatalysis process in 180 min. Figure 7b shows the results of MB dye degradation using the PVDF–BCZTO ceramic composite in the piezocatalysis process. Later in the study, the 10 wt.% BCZTO ceramic–PVDF composite film performance was remarkable, which inactivated ~99.99% of the *E. coli* in 180 min under the influence of piezocatalysis, as shown in Figure 7c.

**Figure 8:**
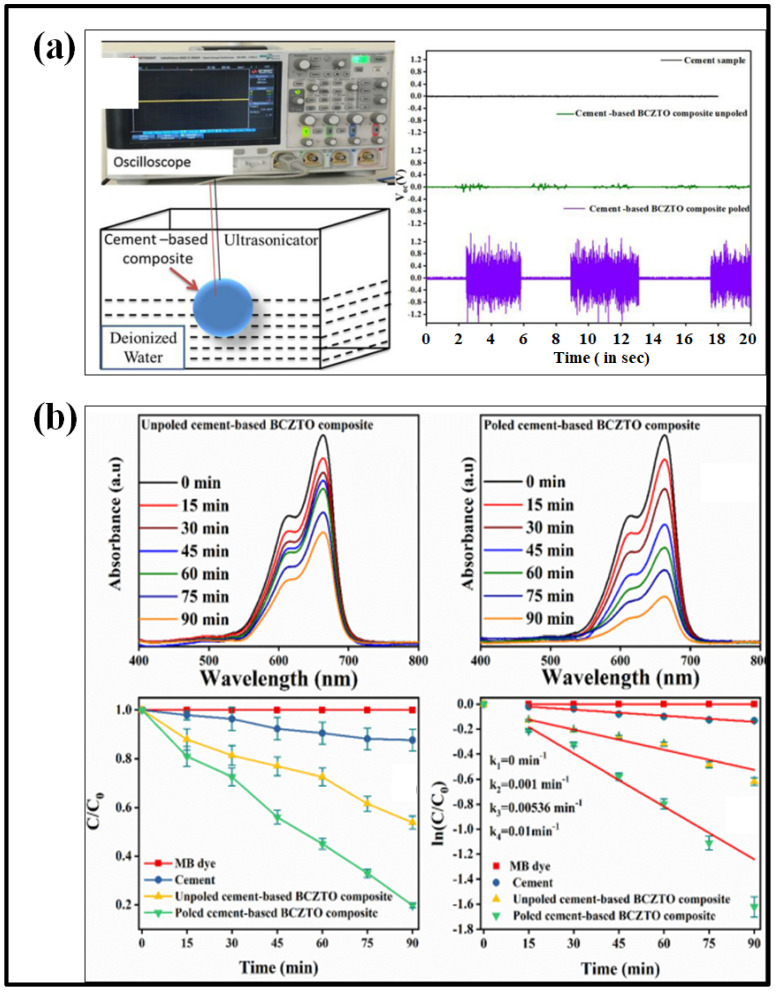
(**a**) Generation of piezoelectric voltage using BCZTO-based cement composite, and (**b**) piezocatalytic activity of unpoled and poled BCZTO-based cement composites for degradation of MB dye. Reproduced with permission from Moolchand Sharma et al., Materials Today Communication; published by Elsevier, 2020 [99].

In the context of its reusability after any catalysis process and being a cost-efficient catalyst, the BCZTO ceramic was mixed with Portland cement in a 50 wt.% proportion and investigated for degrading MB dye via the piezocatalysis process [99]. Cement is one of the most used and common structural materials. Unlike ceramics, which require a specific high-temperature sintering procedure to generate a desired shape, cement-based composites can be cast at ambient temperature in any desired shape and size. In cement-based piezoelectric composites, antibacterial and dye degradation activities were demonstrated when Portland cement was mixed with unpoled and poled BCZTO ceramics using the conventional cement water-hardening procedure. Under ultrasonication, the activity of dye degradation in unpoled and poled composites was examined. Figure 8a,b shows the piezoactivity and degradation capability of the unpoled and poled composite. It was found that the poled cement-based composite was able to completely kill *E. coli* bacteria in 90 min. 

Table 1 shows a comparison between the most common ferroelectric and BCZTO ceramics for multicatalytic activities.

## 4. Future Scope of Ferroelectric BCZTO Ceramic 

It is important to highlight that besides its impressive electrical properties, the BCZTO ceramic has applications in the field of catalysis. However, given the significant advancements in science and technology, coupled with the growing issues caused by human activities, it is crucial to address real-life problems. Therefore, exploring the potential of the BCZTO ceramic for solving real-time issues is a promising avenue that should be addressed promptly. Additionally, as new catalysis processes such as tribocatalysis are emerging [110], it would be worth investigating the feasibility of the BCZTO ceramic as a tribocatalyst. As in AOPs, the synergistic effect produces enhanced catalytic activities, a more synergistic effect of the combined catalysis processes needs to be addressed using the BCZTO ceramic [111].

**Figure 9:**
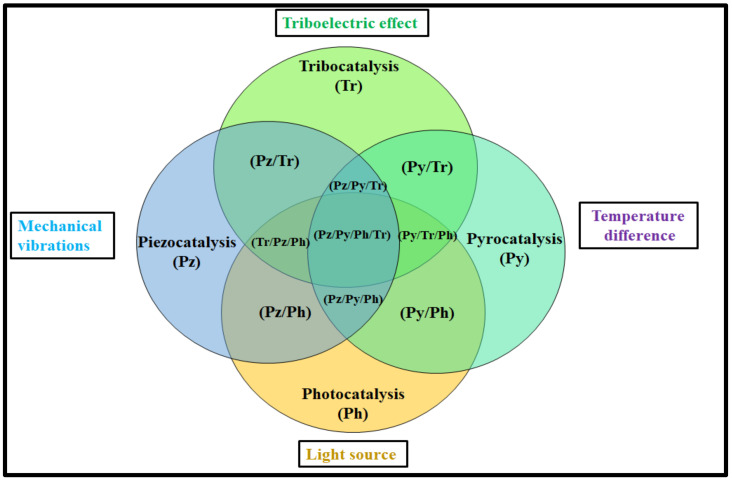
Possible combinations of processes for water remediation using BCZTO ceramic.

Although, some combinations of synergistic effects of the catalysis process have been studied for the BCZTO ceramic, with evolving scientific knowledge, it is conceivable that, in the future, improved catalytic activity could be attained by effectively combining some of the catalysis processes. Figure 9 shows some of the combinations of catalysis/synergistic effects in processes such as pyro/photocatalysis, tribo/photocatalysis, tribo/piezocatalysis, tribo/piezo/photocatalysis, etc., which are not used in BCZTO ceramics.

Furthermore, environmental remediation encompasses more than just addressing contaminated water; it extends to air purification as well. Ferroelectric ceramics, such as BCZTO, are poised to become a significant trend in this domain, showcasing their potential for air purification applications. One promising avenue for the BCZTO ceramic lies in the utilization of electrospinning techniques. Through electrospinning, fibers with diameters ranging from a few nanometers to a few micrometers can be produced, and these fibers contain an embedded ceramic phase [57]. The vast surface area offered by these electrospun fibers make them highly effective in the degradation of pollutants. As a result, exploring the BCZTO ceramic in this context holds great promise for advancing water and air purification technologies.

## 5. Conclusions

The BCZTO ceramic was investigated for its multifunctional catalytic capabilities, including photocatalysis, piezocatalysis, and pyrocatalysis, in the degradation of organic pollutants and disinfection of bacteria in certain instances. Initially, the BCZTO ceramic displays limited reactivity to visible light. However, its photocatalytic activity can be significantly improved by incorporating noble metals such as Ag, doping transition metals such as Fe, and subjection to electric poling. For piezocatalysis, the poled BCZTO ceramic exhibits enhanced performance as the aligned dipoles align with the applied field, leading to a reduced recombination rate. Moreover, when loaded with Ag, the BCZTO ceramic shows further improvement in piezocatalysis. Considering the importance of catalyst reusability, the BCZTO ceramic proves its versatility by retaining its catalytic activity when embedded in materials such as PVDF and cement, making it a flexible candidate. In conclusion, the BCZTO ceramic demonstrates promising multifunctional catalytic activity and holds great potential for applications in water remediation and disinfection due to its ability to efficiently degrade pollutants and disinfect bacteria in various environmental contexts.

## Figures and Tables

**Table 1:** Some known ferroelectric materials and their multicatalytic activities.

**Ferroelectric Materials as Photocatalysts and Their Pollutant Degradation Performances**				
**Catalyst**	**Form**	**Organic Pollutant**	**Source**	**Performance**
BaTiO_3_ [100] Ag-loaded BaTiO_3_ [101] BaTiO_3_ [102] BiFeO_3_ [103]	Nanoparticles (0.07 g) Ag nanoparticles on BaTiO_3_ (0.15 g) Pellet Micro-particles (0.5 g/L)	MB dye (250 mL) (~5 mg/L) RB dye (50 mL) (~10 mg/L) *E. coli* bacteria (10^6^ CFU/mL) Tetracycline (~10 mg/L)	150 W mercury lamp Solar simulator 365 nm UV length Visible light (>420 nm wavelength)	64% in 50 min 100% in 60 min ~90% under UV light in 30 min 69 % in 120 min
**Ferroelectric material as piezocatalysts and their pollutant degradation performance**				
**Catalyst**	**Form**	**Organic pollutant**	**Source**	**Performance**
BaTiO_3_ [75] Bi_4_Ti_3_O_12_ [38] BiFeO_3_ [104] BaTiO_3_ [105]	Nanowires (0.1 g) Nanostructure (0.05 g) Nanosheets (0.05 g) Powder (micron-sized)	RB dye (100 mL) (~5 mg/L) RB dye (100 mL) (~5 mg/L) RB dye (50 mL) (~5 mg/L) Ciprofloxacin (10 mL)	Sonicator (40 kHz, 80 W) Sonicator (40 kHz, 80 W) Sonicator (45 kHz) Sonicator (40 kHz, 70 W)	100% in 60 min 95.7% in 160 min 94.1% in 50 min 85% in 150 min
**Ferroelectric material as pyrocatalysts and their pollutant degradation performance**				
**Catalyst**	**Form**	**Organic pollutant**	**Source**	**Performance**
BaTiO_3_@Ag [106] BiFeO_3_ [107] Ba_0.7_Sr_0.3_TiO_3_@Ag [69] NaNbO_3_ [108] LiNbO_3_ and LiTaO_3_ [109]	Nanofibers Nanoparticles Nanoparticles Nanoparticles Nanoparticles	RB dye (5 mg/L) RB dye (5 mg/L) RB dye (5 mg/L) RB dye (5 mg/L) *E. coli* bacteria inactivation	72 cycles, (30–52 °C) 85 cycles, (27–38 °C) 50 cycles, (20–50 °C) 24 cycles, (23–50 °C) 60 min, (20–45 °C)	92% 99% 90% 96% 95%
**Ferroelectric BCZTO as photo/piezo/pyrocatalyst and its pollutant degradation performance**				
**Catalyst**	**Form**	**Organic pollutant**	**Process/Source**	**Performance**
BCZTO [49] Ag-loaded BCZTO [92] Ag-loaded BCZTO [92] BCZTO-Fe (1%) [84] BCZTO [84] 10 wt.% BCZTO/PVDF film [97] Cement-based BCZTO composite [99]	Pellet Micron-sized powder (0.1 g) Micron-sized powder (0.1 g) Pellet Pellet Pellet Micron-sized BCZTO in PVDF film Poled BCZTO in cement	RB dye (10 mg/L) RB dye (20 mL) MB dye (5 mg/L, 10 mL) MB dye (~5 mg/L) MB dye (~5 mg/L) MB dye (~5 mg/L) MB dye (~5 mg/L) *E. coli* bacteria	Piezocatalysis/ sonicator (40 kHz, 70 W) Photocatalysis/ visible light Piezocatalysis/ sonicator (40 kHz) Photocatalysis/ visible light (15 W, 420 nm LED) Piezocatalysis/ sonicator (40 kHz, 70W) Pyrocatalysis/ 10–45 °C, 200 cycles Piezocatalysis/ sonicator (40 kHz) Piezocatalysis/ sonicator (40 kHz)	90% in 180 min 99% in 40 min 96% in 90 min 94% in 210 min ~78% in 150 min ~92% 91% in 180 min ~99% in 90 min

## Data Availability

The authors confirm that the data supporting the findings of this study are available within this manuscript.
